# Neural Analysis

**Published:** 1892-10

**Authors:** B. Fincke

**Affiliations:** Bureau of Homoeopathic Philosophy, I. H. A.


### NEURAL ANALYSIS.

### Prof. Dr. Gustav. Jaeger's Latest Provings of Homœopathic Potencies.

B. Fincke, M. D.

(Bureau of Homœopathic Philosophy, I. H. A.)

About ten years ago Prof. Dr. Gustav Jaeger, in Stuttgart, surprised the homœopathic world with his investigation of homœopathic high potencies by his method of Neural Analysis. As you know, by a delicate electric chronoscope which shows on its dial as small a quantity of time as a mill second or a thousandth of a second, he measured the time which passes between looking at the beginning of the hand to move and the pressure with the finger by which the movement is stopped.

The distance between the eye and the finger is measured by the number of mill seconds passed over and thus indicates the velocity of the nerve-action, and therefore corresponds to the distance of the hand from the starting point to its stop, by mill seconds. This is called the *nerve-time*. By introducing into the organism medicinal substances, the nerve-time in the subsequent observations is either accelerated or retarded, in other words, they shorten or lengthen the nerve-time. The result of the investigation was very favorable to high potencies which gave the shortest nerve-time. The 4000th centesimal potency gave the highest percentage.

Now lately Dr. Jaeger has been invited by the publisher of the *Allgemein Homœopathische Zeitung* to resume his neural analytical researches, to which he readily responded. The first part of his work is before us, and contains a table which shows the result of his nervo-analytical provings of Kali-carbonicum in potencies from 3–27 decimal and 30–1000 centesimal. The mode of operation is different from the former. The transference of the substance upon the higher potencies was perfected through the whole series in equal vials, which is supposed to mean that they were carried up on the remaining drop according to Korsakoff's method, but no more particulars are given. Of the potency to be proved, one drop was dropped into a glass with about two grammes of distilled water and the whole swallowed at once. Previously observations were taken in health without taking anything, then with as much swallowed distilled water as was used for the analysis, and after that the medicinal fluid was tested. The numbers of the nerve-time of all these observations were taken down chronologically.

The table showing the medicinal action of Kali-carbonicum is remarkable for the result obtained from the numerous measurements. Dr. Jäeger, this time, has taken particular care to analyze the lower decimal potencies which have come into such favorite use against the warnings and advice of Hahnemann. As the time is too short to criticise in particular, we only point out what is of the greatest interest to the Hahnemannian homœopathician, without entering more than necessary upon the reasonings of Dr. Jaeger, which, from his standpoint of materialism, disagree with that of Hahnemann, viz., Dynamism.

The lowest potency tested, the 3d decimal, gives after six minutes a sum of the values of the nerve-time-numbers obtained of —255, which means that the nerve-time during these six minutes has been decreased by 255. Dr. Jaeger draws the conclusion that the action of this low potency, after showing at the end of the first minute the opposite, is a paralyzing effect.

The next potency is the 5th dec. in 7 minutes —251, and the 7th dec. in 10 minutes —80.

From the 9th to the 25th dec. the numbers rise with the opposite effect of increasing the nerve-time, which gradually rises through the

| | | | | | |
|---|---|---|---|---|---|
| 9th | decimal | 5 | minutes | with | + 21. |
| 11th | " | 7 | " | " | +215. |

| | | | | | |
|---|---|---|---|---|---|
| 13th | decimal | 6 | minutes | with | +191. |
| 15th | " | 6 | " | " | +296. |
| 17th | " | 7 | " | " | +441. |
| 19th | " | 7 | " | " | +566. |
| 21st | " | 7 | " | " | +548. |
| 23d | " | 7 | " | " | +669. |
| 25th | " | 7 | " | " | +645. |
| 27th | " | 7 | " | " | +579, to the |
| 30th cent. | " | 8 | " | " | +999. |

This result is remarkable, and that Hahnemann's advice was not without reason when he recommended this potency for treatment in disease and proving in health and for the sake of uniformity of observation. The rise of the numbers from the 23d dec.=11½ centesimal near the limit of potentiation assigned by the anti-Hahnemannian school with the nerve-time of 669 to 999, is enormous and demolishes the scholars of this school completely who contend that there is no action in potencies beyond the 12th centesimal. Not enough, the rise of the nerve-time goes on through the remaining series of centesimal potencies of the

| | | | | |
|---|---|---|---|---|
| 50th | in 10 | minutes | with | +1073. |
| 100th | " 10 | " | " | +1221. |
| 1000th | " 14 | " | " | +1463. |

Nay, not only is the nerve-time increased with every rising potency, but also the duration and intensity of action.

This is what a preliminary examination of the first table furnishes. A more thorough judgment can only be had when the whole work is finished. For the present the great value of the table is in the production of the numbers of nerve-time, which incontrovertibly show that the potentiation of Hahnemann is a fact which nothing can overturn. By bringing the original substance in a suitable way according to the ratio of 1 : 100 with a series of quantities of inert vehicles so that the medicinal force contained in it is distributed over and assimulated by every particle of the vehicle, the medicinal power of the original substance used is multiplied and spread in all directions, and acquires new forces which answer the varying susceptibility of the organism in health and disease.

The question arises what does the increase and decrease of the nerve-time mean in regard to the organism? Dr. Jaeger comes to a different idea than we, reared in the homœopathic school, have imbibed. We take medicine for the sake of proving, and being in good health at the time, the subsequent symptoms of more or less severity we lay to the distunement of the life-force. In other words, we get sick by the medicine we have taken. Now by Neural Analysis we find that by taking medicine in the lowest potencies the nerve-time is retarded, in the higher ones accelerated. Dr. Jaeger, under the impression of his former neuro-analytical investigations and generalizing thereon, concludes that "much substance, heavy substance, too, concentrated substances paralyze, saturate, retard the vital functions, and reversely small quantities, light, volatile, and attenuated substances animate, accelerate the vital motions," and that "every substance disposes over two opposite factors—a retarding and an accelerating one." "How can that be?" The retardation is explained by the inertia of the factor in conformity with mass, weight. Hence heavy substances have slow motion, also large quantities, and concentrated substance has not space enough for moving. The animation and acceleration come from the light and volatile substances. In the place of the mass and weight, with their inertia, the lightness and volatility introduces the conception of velocity as the other factor, and now Dr. Jaeger arrives at the explanation of potentiation, viz., that the more the quantity of the substance diminishes the more increases the velocity of its internal motion as in warming, or it assumes the properties of a volatile substance, and this increases with increasing attenuation, as the table shows.

There is an irreconcilable difference between this theory and the Hahnemannian view. How can that which makes the healthy organism sick at the same time animate him? The idea that what accelerates nerve-action is also animating it—*i. e.*, giving life—increase life—cannot be admitted. That which excites the nerve-system cannot be said to animate it. On the contrary, by increasing the action upon the nerves it accelerates the organic change, and as this implies loss, which is to be repaired, it can only produce the opposite of animation, viz.: loss of vital power.

In regard to the idea that the lower potencies on account of the quantity of substance paralyze the system, because they retard the nerve-action, it does not prove that it deadens this action by retardation. It only shows that there action of the life-force is not as lively as in the higher potencies; but why? It's not easy to say, and we wish to lay the proof of it to the feet of the low potentialists. Besides, the time is too short to enter upon this as interesting as intricate subject.

The line of reasoning of Dr. Jaeger was only mentioned because it leads him to the molecular theory to which he "leans," and makes him call potentiation "the increase of velocity of the interstitial molecular motion." And he threatens the physicists that they shall have to extend their physical theory because the facts go against it, and must prevail if the theory is to go down. But the Doctor is not aware that with this his leaning to the molecular theory goes down, too, and as he progresses in his investigations of potentiation he no doubt will have to give up the standpoint of the old school, which depends upon materialism, and can never comprehend Hahnemann's potentiation if they hitch it on to the molecular theory.

The adherents and professors of this theory have already denounced all potencies above the 12th centes., as being nothing but delusion. The calculations of the greatest men in physical science have not by far reached even that limit. How can the Hahnemannian, with his thirtieth, hundred-thousandths, and million potencies lean upon such a fragile support as the molecular theory? He has to emancipate himself from his thraldom of inert matter.

However, be that as it may, the efforts of our friend, Dr. Jaeger, are of inestimable value, because he furnishes facts and dates in favor of homœopathic potentiation which cannot be silenced down nor controverted, for "numbers prove."

Dr. Jaeger complains of the small encouragement which he had from the homœopathic side ever since he published his immortal investigations ten years ago, and as he undergoes the great labor only in the interest of real science and for the best of Homœopathy which he found himself bound to acknowledge as true, after his diligent search for the truth, every homœopathician ought to express freely his appreciation of his noble efforts and his thanks for what he has done for Homœopathy.

### Discussion.

A motion was made and carried that a letter be sent to Dr. Jaeger by the Corresponding Secretary, expressing the gratification of the Association at the results of his investigation.

Dr. Tompkins—I understand that Dr. James has made some experiments in this line and would like to hear from him as to the nature of the instrument, and also what he thinks of the value of these experiments.

Dr. W. M. James—In regard to the actual practical value of the experiments, I cannot give a satisfactory answer. The work was being prosecuted by Dr. Fellger and was brought to a summary conclusion by his death. We had only experimented about two months, which was not time enough to arrive at any definite conclusions, and whatever results were obtained have now been lost. The instrument consisted essentially of a clock dial and works, the latter moved by a heavy weight. Behind it were two electro-magnets placed opposite to each other and drawing on or affecting an armature which lay between them. There was also a push-button and the instant that it was pushed as well as the instant it was released were accurately recorded on the dial and could be read off without calculation.

Dr. Tompkins—Did the experiments leave an impression on your mind that there was something in neural analysis or not?

Dr. W. M. James—Yes, I believe there is something in it, but it requires an immense amount of training to carry on the experiments accurately.

